# Unraveling the Complexity of Internet Addiction: A Multi-Faceted Perspective from Five Key Studies

**DOI:** 10.3390/jcm14072534

**Published:** 2025-04-07

**Authors:** Silvia Cimino, Luca Cerniglia

**Affiliations:** 1 Faculty of Psychology, Department of Dynamic, Clinical and Health Psychology, Sapienza University of Rome, 00185 Roma, Italy; silvia.cimino@uniroma1.it; 2 Faculty of Psychology, International Telematic University Uninettuno, 00186 Roma, Italy

Internet addiction (IA) is one of the global concerns of our time, and research continues on understanding its psychological, neurobiological, and behavioral bases [1,2,3]. Although not yet officially recognized as a formal psychiatric disorder, its relationship with impulse control disorders and behavioral addictions led to a large amount of scientific debate [4,5,6]. The five studies reviewed in this editorial offer complementary perspectives with respect to different facets of IA, its relationship with personality, resilience, compulsive use of pornography, potentially problematic gaming behaviors, and the difficulties of diagnosis. Combined, they underscore the complexity in properly diagnosing and addressing IA, and the need for a nuanced approach in its understanding and treatment that incorporates individual variations and environmental factors, alongside diagnostic inconsistencies [7,8,9]. These specific studies were chosen due to their specific focus on IA and large resonance in the academic community. In fact, these researchers are among the most viewed and cited in this Journal. The five articles reviewed in this editorial significantly contribute to the discourse regarding the potential official recognition of Internet Addiction (IA) as a formal psychiatric disorder. Specifically, they address critical issues such as diagnostic precision, the validity and utility of existing assessment tools, and the refinement of diagnostic criteria (DSM-5 and ICD-11). By highlighting core symptoms like withdrawal and loss of control, validating robust diagnostic methods, and demonstrating significant associations with recognized psychiatric comorbidities, these studies collectively strengthen the empirical foundation necessary for IA’s potential recognition. Although official recognition involves broader consensus within clinical and psychiatric communities, these articles certainly add substantial evidence, highlighting critical diagnostic and clinical parameters essential for future consideration and potential formal classification of IA.

The study by Cho Rong Nam et al. [10] explores the complex relationship between individual resilience and internet addiction (IA) in groups of adolescents, focusing on sex differences. Internet addiction has been increasingly recognized as a behavioral disorder with negative outcomes for mental health, for example, anxiety, depression, impulsivity, and social isolation. The aim of this study was to verify whether or not resilience plays a protective role against IA and if and how it interacts with behavioral inhibition and activation systems (BIS/BAS) in boys and girls. The sex-divergent effects described in the article by Cho Rong Nam et al. [10] provide meaningful insights into IA’s nuanced development pathways and risk factors. Notably, their findings indicate that resilience plays a protective role predominantly for female adolescents, suggesting distinct coping strategies and psychological vulnerabilities across sexes. Girls typically employ more active, problem-focused coping mechanisms (e.g., seeking social support), making resilience a stronger protective buffer against IA. Conversely, boys may exhibit more impulsivity and avoidant coping strategies, potentially mitigating the protective effect of resilience. This critical insight emphasizes the need for gender-specific preventive and therapeutic interventions, recognizing distinct risk profiles and psychological mechanisms across sexes.

The researchers draw on Gray’s neuropsychological theory, which posits that human behavior is regulated by two fundamental neural mechanisms: the behavioral inhibition system (BIS), associated with sensitivity to punishment and avoidance of negative outcomes, and the behavioral activation system (BAS). The latter is thought to be linked to reward-seeking and impulsive behaviors. Previous research has shown that high BAS activity is frequently connected with addictive behaviors, including substance use disorders and/or gambling. Nevertheless, the role of BIS in addiction remains less clear. Moreover, some studies suggest that low BIS levels exacerbate addictive tendencies, while others indicate that both high BIS and high BAS can contribute to internet addiction through distinct pathways. The study recruited 519 middle-school students in South Korea (268 boys and 251 girls), who filled out validated self-report questionnaires measuring a series of variables such as internet addiction, BIS/BAS levels, depression, anxiety, impulsivity, anger, and resilience. Using statistical models, the researchers tested whether resilience moderates the relationship between BIS/BAS and internet addiction through the mediation of clinical variables.

The above findings suggest that resilience plays a protective role in the development and maintenance of internet addiction, but its effects are sex-dependent. While both boys and girls showed similar mediation pathways in which BIS and BAS influenced internet addiction through depression, anxiety, impulsivity, and anger, the moderating effects of resilience were found to be significant only in girls. In other words, resilience buffered the impact of psychological distress on internet addiction in female adolescents, but not in males.

These results are consistent with previous literature suggesting that girls generally score higher on resilience measures, while tending to use more constructive coping strategies (e.g., seeking social support and engaging in problem-solving behaviors). Boys, on the other hand, exhibit higher levels of avoidant coping and may be more prone to impulsivity-driven internet use, making them less responsive to the protective effects of resilience.

The study raises important questions about the role of digital environments in shaping adolescent mental health. With increased access to the internet and social media by young individuals, understanding the mechanisms that drive problematic internet use is crucial for developing effective prevention and intervention strategies. Further research should explore longitudinal patterns of internet addiction, examining how resilience develops over time and whether targeted interventions can enhance its protective effects in at-risk populations.

Alarcón et al. [11] explore the topic of problematic online pornography use (POPU) as a form of behavioral addiction, placing it within the broader framework of compulsive sexual behavior (CSB) and hypersexual disorder. The paper recognizes the increasing scholarly attention given to online pornography addiction; however, it also highlights the current lack of a standardized diagnostic framework and the difficulty in differentiating pathological from non-pathological consumption. One of the main issues in the study of online pornography addiction is conceptual ambiguity. While some researchers categorize compulsive sexual behavior under impulse control disorders, others suggest that it fits more appropriately within behavioral addiction models, akin to gambling disorder. The American Psychiatric Association (APA) has not yet included online pornography addiction in the DSM-5, although the label of compulsive sexual behavior disorder (CSBD) was recently incorporated in the ICD-11 under impulse control disorders. This classification remains controversial, as many scholars argue that POPU aligns more closely with substance use disorders, due to the dopaminergic reward mechanisms involved and the potential for tolerance and withdrawal symptoms.

The authors systematically compiled findings on the epidemiology, clinical manifestations, neurobiological evidence, and treatment approaches for POPU. They highlight that internet pornography use has surged in recent decades, thanks to the “Triple A” model—accessibility, affordability, and anonymity. These three factors make pornography consumption extremely easy and pervasive, leading to greater opportunities for problematic consumption patterns to emerge. The increasing consumption of pornography is particularly notable among young males, and some evidence suggests that early exposure to online pornography may influence sexual development and behavior patterns.

From a professional standpoint, problematic internet pornography consumption is frequently linked to adverse psychological and interpersonal outcomes. Research indicates that people with POPU experience elevated levels of anxiety, depression, social dysfunction, and discomfort associated with their intake. A multitude of users encounter a lack of control regarding their pornography intake, facing unsuccessful endeavors to reduce usage while having relational challenges, occupational issues, and emotional turmoil. Furthermore, an increasing volume of research suggests a correlation between excessive pornography use and sexual dysfunctions, notably erectile dysfunction (ED) and reduced sexual satisfaction with actual partners. This has prompted several academics to propose that online pornography establishes an inaccurate benchmark for sexual satisfaction, teaching consumers to choose fake digital stimuli over genuine interpersonal connections. According to the paper’s examination of neurobiological data, POPU is similar to other behavioral addictions and drug use disorders. According to neuroimaging research, compulsive pornography users have increased reactivity in reward-processing brain areas such as the prefrontal cortex, amygdala, and ventral striatum. These patterns are comparable to those seen in drug and gambling addiction. According to certain research, tolerance occurs when people need more and more intense or unique information to achieve the same degree of excitement, which encourages obsessive consumption. It is still up for dispute; nevertheless, if withdrawal symptoms manifest similarly to those of drug addiction, some users may not exhibit the typical symptoms of withdrawal, while others feel mental anguish, cravings, and irritability when refraining.

The analysis also highlights the issue of self-perception among those who believe they have a pornographic addiction. According to some research, POPU’s detrimental effects on mental health are largely caused by subjective perceptions of addiction rather than empirical indicators of compulsive behavior. That is, even when their actual consumption patterns do not fit the clinical criteria for addiction, those who feel that their use of pornography is excessive or embarrassing are more likely to suffer from psychological distress. This aspect of self-perception makes it more difficult to set precise diagnostic cutoff points and raises the possibility that moral, religious, and cultural convictions might occasionally be a contributing cause to suffering.

Although some case reports indicate that SSRIs (selective serotonin reuptake inhibitors) and opioid antagonists (e.g., naltrexone) may help reduce cravings in people with compulsive pornography use, the review finds little empirical support for pharmacological interventions as treatment approaches. Nonetheless, the most often suggested treatment for behavioral addictions, including POPU, is still cognitive-behavioral therapy, or CBT. According to a number of studies, acceptance and commitment therapy (ACT) and mindfulness-based therapies may also be beneficial in assisting people in controlling their impulses, reducing feelings of shame, and managing cravings.

Ryu et al. [12] investigated the Diagnostic Interview for Internet Addiction (DIA) as a semi-structured technique for evaluating Internet Gaming Disorder (IGD) in Korean teenagers. Since IGD was added to Section III of the DSM-5 and ICD-11, there has been an increasing awareness of the need for precise diagnostic instruments to detect and assess the disorder’s severity. Based on their IA symptoms and associated psychological characteristics, the study assesses the DIA’s psychometric qualities and looks at how well it can distinguish between teenagers who are mildly, moderately, or severely at risk, as well as those who are addicted.

The study was conducted on 103 Korean adolescents aged 13–18 years who were divided into three groups: mild risk (0–2 DIA symptoms), moderate risk (3–4 symptoms), and addicted (5 or more symptoms). The DIA consists of 10 items, assessing key IGD criteria such as cognitive salience, withdrawal, tolerance, difficulty in regulating use, loss of interest in other activities, persistent use despite negative consequences, deception about gaming habits, use of gaming to relieve negative emotions, interference with daily life, and craving. Unlike self-report questionnaires, the DIA also includes caregiver interviews, offering a more comprehensive assessment of gaming behavior.

The study found that higher DIA scores were significantly correlated with higher levels of internet and smartphone addiction, anxiety, depression, impulsivity, aggression, and stress, as well as lower self-esteem. Adolescents in the moderate risk and addicted groups exhibited significantly greater psychological distress compared to the mild-risk group. This confirms that IGD is associated with internalizing (e.g., depression, anxiety) and externalizing (e.g., aggression, impulsivity) psychopathologies.

One of the key findings was that low persistence, self-directedness, and cooperativeness (measured using the Junior Temperament and Character Inventory) were significantly associated with IGD severity. Adolescents in the mild risk group demonstrated greater self-discipline and goal-oriented behavior, whereas those in the addicted group had difficulty maintaining long-term goals and controlling impulsive tendencies. This aligns with previous research suggesting that IGD is closely linked to impulsivity and poor executive functioning [13,14].

The study validates DIA as a reliable and valid tool for screening and diagnosing IGD in adolescents. Unlike self-report measures, the semi-structured interview format allows clinicians to cross-check adolescent responses with those of their caregivers, reducing the risk of underreporting due to social desirability bias. Given that the moderate-risk and addicted groups exhibited similar levels of psychological distress, the authors suggest that intervention should begin when adolescents meet at least three IGD criteria, rather than waiting for them to reach the addiction threshold.

The study by Jo et al. [15] examines the differences between the DSM-5 and ICD-11 diagnostic criteria for Internet Gaming Disorder (IGD) and their respective clinical implications. The inclusion of IGD in Section III of the DSM-5 and Gaming Disorder (GD) in the ICD-11 has led to ongoing debates regarding the most appropriate criteria for diagnosing problematic gaming. This study aims to compare the clinical characteristics, gaming behavior patterns, and psychiatric comorbidities associated with IGD based on these two classification systems.

The research was conducted using clinical cohort data collected in South Korea, assessing 188 adolescents through semi-structured diagnostic interviews conducted by psychiatrists and psychologists. One of the key distinctions between the DSM-5 and ICD-11 criteria lies in their diagnostic thresholds and emphasis. The DSM-5 defines IGD as meeting at least five out of nine criteria, which include preoccupation, withdrawal, tolerance, continued use despite negative consequences, deception, and escapism. In contrast, the ICD-11 criteria for GD require three core symptoms: impaired control over gaming behavior, increased priority given to gaming over other activities, and continuation or escalation of gaming despite negative consequences. While the DSM-5 criteria capture a broader range of problematic gaming behaviors, the ICD-11 criteria focus on more severe cases characterized by clear functional impairment.

The findings indicate that the DSM-5 criteria are more inclusive, diagnosing a significantly larger number of individuals compared to the ICD-11. Among the 188 participants, 73 (38.8%) met the DSM-5 IGD criteria, whereas only 12 (6.4%) met the ICD-11 GD criteria. Notably, all individuals diagnosed under the ICD-11 criteria also met the DSM-5 criteria, confirming that the ICD-11 imposes a stricter threshold for diagnosis. The study further examined psychiatric comorbidities associated with IGD and found that adolescents diagnosed under both DSM-5 and ICD-11 exhibited higher levels of depression, conduct disorder (CD), and oppositional defiant disorder (ODD) compared to those who met only the DSM-5 criteria. The group meeting both DSM-5 and ICD-11 criteria showed the highest prevalence of comorbid psychiatric conditions, suggesting that the ICD-11 may be more effective in identifying severe cases where IGD is closely linked to emotional and behavioral dysfunction. Attention-deficit/hyperactivity disorder (ADHD) was the most commonly observed comorbidity across all IGD groups, aligning with previous research indicating that impulsivity and executive dysfunction contribute to excessive gaming behavior. However, ADHD prevalence did not differ significantly between the groups, suggesting that while ADHD increases vulnerability to IGD, its severity does not necessarily differentiate moderate cases from severe ones. Additionally, the study explored gaming behavior patterns, revealing that individuals diagnosed under both DSM-5 and ICD-11 criteria had started gaming at an earlier age, spent more time gaming per day, and engaged in multi-platform gaming more frequently. Adolescents diagnosed with ICD-11 GD spent an average of 477 min per day gaming on weekends, compared to 400 min for the DSM-5 IGD group and 361 min for the normal group. This suggests that the ICD-11 criteria may be more effective at identifying individuals whose gaming behavior is extreme and significantly interferes with daily life.

Moreover, the study found that craving and deception about gaming habits were more prevalent in individuals diagnosed under the DSM-5 criteria compared to those diagnosed under the ICD-11. This finding reinforces the idea that the DSM-5 criteria encompass a wider range of behavioral patterns, including subjective distress, whereas the ICD-11 focuses on observable, functional impairments. The results provide critical insights into how different diagnostic systems influence the identification of gaming disorders. The ICD-11 criteria appear to be more stringent, capturing only the most severe and functionally impaired cases, whereas the DSM-5 criteria encompass a broader range of problematic gaming behaviors. As is known, internet addiction lacks formal, universally agreed-upon diagnostic criteria. However, the reviewed articles collectively discuss and critique existing criteria applied to specific subtypes of IA, notably Internet Gaming Disorder (IGD) and problematic online pornography use (POPU). Specifically, the DSM-5 criteria for IGD (which require five out of nine symptoms, including withdrawal, loss of control, tolerance, and negative consequences) and the stricter ICD-11 criteria (focused on impaired control, prioritization of gaming, and significant functional impairment) are comprehensively analyzed and compared in the reviewed studies. Additionally, the discussion in the POPU context addresses how behavioral addiction criteria (like compulsivity, impaired control, and negative consequences) parallel substance addiction frameworks. These detailed explorations effectively demonstrate how general addiction criteria might be adapted and applied to IA broadly, underscoring essential diagnostic elements such as loss of control, withdrawal, and negative life impacts.

These findings have important implications for clinical practice, policy, and future research. The stricter diagnostic criteria of the ICD-11 may help prevent over-diagnosis but could also overlook early-stage IGD cases that might benefit from intervention. In contrast, the DSM-5 criteria may be better suited for identifying individuals experiencing subjective distress and psychological symptoms associated with problematic gaming. The study highlights the need for evidence-based treatment guidelines tailored to different severity levels, ensuring that both early-stage and severe cases receive appropriate interventions.

The study by Pontes et al. [16] further examines the clinical validity of IGD diagnostic criteria and evaluates whether current frameworks accurately identify disordered gaming behavior. This research employs a tree-based statistical model to assess which IGD criteria carry the most diagnostic weight and how they influence the probability of receiving an IGD diagnosis. Given the ongoing debate on whether certain symptoms, such as withdrawal and tolerance, should be weighted more heavily or whether a more inclusive approach should be used, the study seeks to determine which symptoms are most predictive of disordered gaming.

The researchers analyzed a large sample of 3377 online gamers, primarily young adults, using the Internet Gaming Disorder Scale—Short-Form (IGDS9-SF), a psychometric instrument that measures all nine DSM-5 IGD criteria. The study applied Conditional Inference Tree (Ctree) analysis, a machine learning algorithm that identifies key patterns in diagnostic classification. By analyzing the symptom endorsement patterns, the study sought to determine which symptoms are most indicative of IGD and which may be less relevant in clinical assessments.

The results indicate that not all IGD criteria contribute equally to a diagnosis. The most predictive symptoms of IGD were withdrawal, loss of control, and negative consequences. Participants who reported withdrawal symptoms when unable to game were significantly more likely to meet the criteria for IGD. Those who endorsed an inability to regulate their gaming behavior had a 77.77% increased probability of being diagnosed with IGD. Additionally, the endorsement of withdrawal, loss of control, and negative consequences collectively increased the likelihood of IGD by 26.66%, emphasizing that negative life impacts should be considered a core criterion. Preoccupation with gaming was commonly reported but was found to be a weaker predictor of disordered gaming unless combined with withdrawal symptoms.

Conversely, other IGD criteria, such as tolerance, deception, and gaming as a form of escapism, were found to be less predictive of severe IGD cases. Although these criteria were present in some individuals with problematic gaming behaviors, they were not as critical in distinguishing disordered gaming from highly engaged gaming. A significant contribution of this study is its critique of the DSM-5 and ICD-11 diagnostic approaches. The DSM-5 framework applies a five-out-of-nine symptom threshold, meaning an individual can be diagnosed with IGD based on any five symptoms. However, the findings suggest that weighing all symptoms equally may not be clinically appropriate, as some symptoms, such as withdrawal and loss of control, are stronger indicators of severe dysfunction than others, such as tolerance and escapism.

These findings have significant implications for clinical practice and future research. The study suggests that future revisions of IGD diagnostic criteria should prioritize withdrawal, loss of control, and negative consequences, while reconsidering the relevance of tolerance and deception in clinical assessments. Given that some individuals exhibit mild symptoms without severe impairment, clinicians should consider a staged approach to intervention, offering early support for at-risk individuals rather than waiting for full-blown addiction to develop [17,18]. Additionally, the study highlights that some gamers self-identify as addicted despite not meeting clinical criteria, suggesting that subjective distress may play a role in problematic gaming behaviors. Future research should explore whether self-perceived addiction influences mental health outcomes and treatment-seeking behavior.

## 1. Conclusions

The studies reviewed provide a multifaceted perspective on the complexities of Internet Addiction (IA) and related behavioral disorders, reinforcing the need for nuanced diagnostic and intervention approaches. The reviewed articles can be considered key studies because they offer a comprehensive analysis of Internet Addiction (IA), addressing its psychological, behavioral, and neurobiological dimensions, emphasizing the need for nuanced understanding and effective diagnosis and treatment. Although the reviewed studies investigate distinct behaviors (general internet addiction, gaming, and online pornography consumption), these manifestations share core addictive features, such as compulsivity, impaired control, escalation of usage (tolerance), and significant negative impacts on personal, social, or occupational functioning. Additionally, overlapping psychological correlates (e.g., impulsivity, depression, anxiety) and neurobiological underpinnings (e.g., enhanced reactivity in reward-related neural circuits) suggest substantial intersectionality among IA subtypes. Consequently, future research would benefit from integrated models exploring shared vulnerability factors and treatment targets across different IA manifestations, as the current separation of subtypes may obscure commonalities in addictive pathways and therapeutic opportunities. Furthermore, these studies employ rigorous methodological approaches, including systematic reviews, advanced statistical models such as moderated mediation analysis and Conditional Inference Trees, and validated diagnostic tools, ensuring robust and reliable findings.

The findings highlight the interplay between psychological factors, neurobiological mechanisms, and individual resilience in shaping the trajectory of IA, particularly among adolescents. Cho Rong Nam and colleagues demonstrated the protective role of resilience in mitigating IA-related distress, particularly among female adolescents, underscoring the importance of gender-specific interventions. Meanwhile, Alarcón et al. [11] examined problematic online pornography use (POPU), revealing its potential alignment with behavioral addiction models, yet emphasizing the conceptual ambiguity surrounding its classification.

The diagnostic complexities of IA were further explored by Ryu and colleagues, who validated the Diagnostic Interview for Internet Addiction (DIA) as a robust tool for assessing Internet Gaming Disorder (IGD). Jo et al. [15] highlighted the disparities between DSM-5 and ICD-11 diagnostic criteria, demonstrating how different frameworks influence the identification and treatment of IGD. Pontes et al. (2019) [16] further refined IGD diagnosis by identifying core predictive symptoms, advocating for a reevaluation of existing criteria. Collectively, these studies underscore the need for precision in IA diagnosis and treatment, advocating for evidence-based strategies that account for individual variability and evolving digital landscapes.
